# An Analysis of Students’ Attitudes Toward Artificial Intelligence—ChatGPT, in Particular—In Relation to Personality Traits, Coping Strategies, and Personal Values

**DOI:** 10.3390/bs15091179

**Published:** 2025-08-29

**Authors:** Simona Maria Glaveanu, Roxana Maier

**Affiliations:** Faculty of Psychology, Ecological University of Bucharest, 061341 Bucharest, Romania; roxana.maier@ueb.education

**Keywords:** attitudes, cognitive component, affective component, behavioral component, ChatGPT, university students, adapted scale

## Abstract

The general objective of this research was to investigate the attitudes of Bucharest students toward artificial intelligence (AI)—in particular, ChatGPT—in relation to their personality traits, coping strategies, and personal values to identify psychosocial approaches for students’ effective reporting toward this AI product. As there was no instrument validated and calibrated on Romanian students, the scale constructed by Acosta-Enriquez et al. in 2024 was adapted to students from Bucharest (N = 508). Following the item analysis, the adapted scale was reduced to 16 items, and, following the factor analysis (EFA–0.81 < α < 0.91), the structure with three factors (cognitive, affective, and behavioral components), explaining 53% of the variation in Bucharest students’ attitudes toward ChatGPT, was maintained considering the results of the confirmatory factor analysis—CFA (χ^2^(79) = 218.345, *p* < 0.001; CMIN/DF = 2.486; CFI = 0.911; TLI = 0.900; RMSEA = 0.058 (90% CI: 0.50–0.065). The present study showed that 85.53% of the research subjects used ChatGPT at least once, of which 24.11% have a positive/open attitude toward ChatGPT, and that there are correlations (*p* < 0.01; 0.23 < r^2^ < 0.50) between students’ attitudes toward ChatGPT and several personality traits, coping strategies, and personal values. It also proves that the three components of the attitude toward ChatGPT (cognitive, affective, and behavioral) are correlated with a series of personality traits, coping strategies, and personal values of students. Although the general objective was achieved and the adapted scale has adequate psychometric qualities, the authors propose in future studies to expand the group of subjects so that the scale can be validated at the level of the Romanian population. In this research, at the end, several concrete approaches are proposed for the effective reporting of students toward this AI product, which, beyond the ethical challenges, also recognizes the benefits of technology in the evolution of education.

## 1. Introduction

Education is a constantly changing process. Innovation in education must keep pace with the evolution of technology, and the introduction of new technologies, such as artificial intelligence (AI), always raises new challenges (Bae et al., 2024; H. P. Hsu et al., 2024; Michel-Villarreal et al., 2023).

The European Commission (2022) published a document entitled „Ethical guidelines for teachers on the use of artificial intelligence and data generated by it in teaching and learning”. It addressed how AI is used in schools to support the efforts of teachers and students and to support administrative tasks in educational environments. Those guidelines were part of the „Digital Education Action Plan (2021–2027)” and were developed by a dedicated Commission expert group, which brought together a wide range of practitioners from the world of education and training, academia, the private sector, and international organizations (European Commission, 2022).

In the specialized literature, some authors have analyzed the impacts of AI on the mental development of children and adolescents, identifying negative correlations between AI and the social adaptability of adolescents (H. P. Hsu, 2025; T. C. Hsu & Hsu, 2025; Lai et al., 2023) and between the students’ excessive dependence on AI software systems and its effect on their cognitive abilities, including decision making, critical thinking, and analytical reasoning (Bae et al., 2024; H. Hsu, 2023).

Overconfidence in AI occurs when users accept AI-generated recommendations without questioning them, leading to errors in task performance and decision-making (Zhai et al., 2024). Other authors studied the negative consequences of AI in the context of higher education to find strategies to effectively integrate AI into the study process (Hutson et al., 2022).

Many studies on AI have been based on concepts about the use of technology in educational settings, which have been approached considering various models:

- The technology acceptance model (TAM), which states that new technology is more easily accepted if it is perceived as being easy to use—a concept that provides insights into the factors that influence students’ acceptance of new technologies (Davis, 1986; Yilmaz et al., 2023; Zhai et al., 2024);
- The self-determination theory (SDT), which identifies the intrinsic and extrinsic motivations that lead to the use of new technologies (Ng et al., 2012; Annamalai et al., 2025);
- The cognitive load theory (CLT), which provides explanations regarding the analysis of cognitive benefits and challenges associated with technological assistance in academic tasks (O. Chen, 2015; Lo, 2023).

Studies on students’ positive attitudes toward AI have shown that a high level of subjective knowledge about this type of software and computer use are correlated factors but are also predictors of that attitude (Kaya et al., 2022), and AI learning anxiety correlates with a negative attitude toward AI (Y. M. Wang et al., 2022; C. Wang et al., 2024b). At the same time, studies examining teachers’ attitudes and anxiety toward artificial intelligence have shown that there is a negative relationship between open attitudes toward artificial intelligence and teachers’ anxiety (Akçaba et al., 2024).

The emergence in November 2022 of rapidly evolving AI software called ChatGPT, which was able to synthesize online available data and communicate them in a conversational way, has been challenging the education system, as the AI software had the ability to solve different tests and write the essays required of students (Abramson, 2023). ChatGPT was quickly adopted by both students and researchers and was appreciated for its ability to engage in human-like conversations, answer questions on a wide range of topics, and assist with various tasks, including writing, programming, and problem-solving (Hartley et al., 2024).

The integration of AI into the educational environment has sparked significant debate about its benefits and potential challenges (Yilmaz et al., 2023), and the rapid adoption of ChatGPT in academic environments has raised concerns about its impact on learning, research, and academic integrity (Nemt-allah et al., 2024). While some studies have highlighted the potential of ChatGPT to improve literature reviews, research assistance, and writing support (Abramson, 2023), others have expressed concern about the over-reliance on AI-generated content and its implications for critical thinking and original studies (Zhai et al., 2024). This perspective emphasizes the need to fully understand how students use ChatGPT and adhere to ethical standards in its use in academic work (Nemt-allah et al., 2024).

Teachers have been experimenting with the software, recognizing that ChatGPT could be a useful tool for preparing students for the real world, where critical thinking is more important than the simple memorization of concepts (Abramson, 2023); however, they draw attention to the need to consider ethical standards, as they would when integrating other technologies into teaching.

AI has many useful applications in instruction and research, and, to compensate for the proliferation of the writing it has generated and the unethical use of ChatGPT, teachers and institutions have been experimenting with and establishing rules to ensure that both students and teachers maintain academic integrity but also to use programs to detect the use of AI text generators (Belair, 2023).

Other studies, starting from investigating what makes students use AI and complementing quantitative data with qualitative data (obtained based on interviews), provide new insights for university management on building effective strategies for applying AI technology (C. Wang et al., 2024b).

The analysis of the relationship between students’ emotional stability and attitudes toward AI revealed positive correlations, and it was found that neuroticism could influence the perceived practicality of technologies (Kaya et al., 2022; C. Wang et al., 2024a). Studies have also indicated that emotional stability mitigated problematic and pathological internet and social media use (Hawi & Samaha, 2019). Regarding extraversion as a personality trait, some studies have shown that it can boost students’ acceptance of technology (Kaya et al., 2022), while others have found a negative association between extraversion and attitudes toward AI, indicating that the more introverted the student, the more positive their perception of AI (Schepman & Rodway, 2022).

The presence of stress can significantly affect the academic performance and general well-being of students; therefore, it becomes necessary to use effective stress management strategies (Tuanany & Nurdianto, 2024). Students under time pressure in completing projects resort to ChatGPT more frequently, which can be associated with the use of ChatGPT as an active stress coping strategy (Azeem & Abbas, 2025; Tuanany & Nurdianto, 2024).

At the same time, students with difficulties in planning, emotional regulation, or completing tasks tend to use AI (including ChatGPT) as support for active academic coping focused on the problem and on reducing negative emotions and anxiety (Klarin, 2024). However, excessive dependence on ChatGPT can affect the development of cognitive skills (Azeem & Abbas, 2025; Klarin, 2024). As a result, understanding coping strategies and the factors that influence them is essential for promoting effective stress management among students (Tuanany & Nurdianto, 2024), and through digital education focused on developing critical AI use skills, students will be able to integrate ChatGPT in a sustainable way for their learning while respecting ethical standards in academic activity.

Students’ morality as a personal value in their compliance with academic ethics in the use of ChatGPT has been analyzed in several studies showing that students’ personal morality leads them to experience regret when they violate academic integrity through unethical use of ChatGPT (Elbaz et al., 2024) or anxiety related to the use of AI in order not to violate ethical standards (Zhu et al., 2024). Along similar lines, other studies have analyzed the relationship between students’ intention to use AI and their perceived utility value from the perspective of costs (ethical integrity) and benefits (on academic outcomes), showing that students who use ChatGPT more frequently perceive a higher utility value of it (Rruplli et al., 2024).

These studies bring to the forefront the importance of in-depth analyses of students’ personal values (considering other types of personal values beyond morality and utility), along with coping strategies and personality traits in students’ balanced reporting on AI and its products, laying the foundations for the formation/development of sustainable attitudes toward ChatGPT.

Previous research explored various aspects of AI assistance in education, including effects on higher education (Schepman & Rodway, 2022; Belair, 2023; Acosta-Enriquez et al., 2024; Hartley et al., 2024); however, there are only a few studies that propose validated instruments that can assess the multidimensional nature of ChatGPT use, especially among students. This fact diminishes the ability to measure and quantitatively analyze the impact of ChatGPT on education, making it difficult to develop management strategies or the ethical use of ChatGPT (Nemt-allah et al., 2024).

Starting from this need mentioned at the international level, but also from other concepts from the specialized literature and from the provisions and recommendations of the National Strategy in the Field of Artificial Intelligence 2024–2027 issued by the Ministry of Research, Innovation, and Digitization, the objectives of the present research were formulated.

## 2. Materials and Methods

The general objective of the research was to investigate the attitude toward artificial intelligence—specifically, ChatGPT—and its relation to some psychosocial variables (personality traits, coping strategies, and personal values) among students in Bucharest, Romania, to identify, beyond the specially dedicated technology management programs, psychosocial approaches for students’ effective reporting toward this AI product.

- Specific objectives

As no diagnostic tool has been built or adapted for the Romanian population, the first specific objective of this research aimed at adapting and using a scale built for and validated on another population that also had adequate psychometric characteristics; those criteria were met by the scale developed by Acosta-Enriquez et al. (2024), and adapting it to the particularities of students from Bucharest, then subsequently analyzing the psychometric characteristics of the new form of the scale.

The second objective involved investigation of the relationships between the attitude of students toward ChatGPT and certain psychosocial variables (e.g., personality traits, coping strategies, and personal values) to identify psychosocial approaches for students’ effective reporting toward this AI product in the university environment.

- Participants and procedure

The subjects of the research were 508 students and master’s students from eleven faculties from Bucharest, Romania (383 female and 125 male), aged between 20 and 49 years (M = 33.66; AS = 10.13), from the Faculty of Psychology (N = 67), the Faculty of Communication Sciences (N = 45), the master’s course in Cognitive Psychodiagnosis and Psychological Counseling (N = 52), the Faculty of Administration and Business (N = 42), the Faculty of Administration and Public Management (N = 31), the Faculty of Foreign Languages (N = 35), the Faculty of Medicine (N = 64), the Faculty of Automatics and Computers (N = 67), the Faculty of Engineering (N = 54), the Faculty of Construction (N = 20), and the Faculty of Agriculture (N = 31).

The selection of participants was carried out by the pseudo-random (convenience) sampling method, which ensured acceptable representativeness for the student population in Bucharest.

The subjects participated voluntarily, being recruited by sharing a Google Forms link, through which they completed the research questionnaires without a time limit after they had provided informed consent regarding the purpose of the research, the authors, and the anonymization of results.

- Instruments

Four instruments were used for the psychological evaluation of the students from the research group. The latter three within the following list are part of the computerized psychological evaluation platform CAS++ (developed by Cognitrom), which has been validated and benchmarked in the Romanian population (which shows that, from a statistical point of view, they can be used with confidence in assessing psychosocial variables: personality, coping strategies, and personal values).

1. The Attitude toward ChatGPT Identification Scale (Acosta-Enriquez et al., 2024).

To identify the attitudes toward ChatGPT of students from Bucharest, Romania, this scale was adapted accordingly.

The original scale had 40 items and was constructed by integrating the concepts of Mitcham (1994) as cited in Acosta-Enriquez et al. (2024) regarding the cognitive, affective, and behavioral components of the attitude toward technology and the integrated model of Svenningsson et al. (2022) as cited in Acosta-Enriquez et al. (2024) regarding the influence of the first two components on the behavioral ones. It was calibrated on 595 Peruvian students from 6 public and private universities, and it has psychometrically appropriate characteristics (from a statistical point of view), as the items of the scale had a very good internal consistency, with the Cronbach’s Alpha coefficients of the three components between 0.84 and 0.96, which indicates the fidelity of the scale.

The scale was adapted to 508 students from Bucharest, Romania, and it contains 16 items, as described in Table 1. The information about its psychometric characteristics is detailed in the result analysis and interpretation section.

2. The CP5F personality questionnaire was designed by Albu in 2008 based on the model of the FFPI (Five-Factor Personality Inventory) designed by Hendriks in 1997 (Cognitrom, 2024b). The CP5F contains 130 items and is meant to evaluate the five super factors of the Big Five model (Extraversion; Emotional stability; Conscientiousness; Kindness; Autonomy) but also includes a scale (called Social desirability) for identifying people whose answers are not in accordance with reality, whether because they want to create a favorable image of themselves, they answer randomly, or they want to appear different from the rest of the people. The Cronbach’s Alpha coefficient values range between 0.61 and 0.76, indicating the fidelity of the scales of the instrument.
3. The Cognitive–Emotional Coping Questionnaire (CERQ) was designed by Garnefski et al. and translated and adapted for the Romanian population by Perțe and Țincas (coordinators) in 2010 (Cognitrom, 2024a). CERQ is a self-assessment questionnaire that measures the cognitive coping strategies of adults and has 36 items that refer exclusively to what a person thinks, rather than what they actually do when living through threatening or stressful life experiences. The CERQ evaluates the following nine cognitive coping strategies: Acceptance; Self-blame; Rumination; Positive refocusing; Refocusing on planning; Positive reappraisal; Putting into perspective; Catastrophizing; and Blaming others. The internal consistency of the items and the fidelity of the scales is reflected by Cronbach’s Alpha coefficients between 0.54 and 0.73.
4. The Evaluation of Values Questionnaire—v21 is a 21-item questionnaire adapted from the 36 items included in the CCP (Cognitive Career Planner)—a platform developed by Miclea et al. (2013) as cited in Cognitrom (2024c). The 21 items are distributed into 7 scales, with each scale evaluating a personal value (Professional recognition; Authority; Social relations; Autonomy; Security; Compliance with the rules; and Challenge). The 21-item questionnaire was standardized using a sample of 609 persons; the internal consistency of the scales was proven by Cronbach’s Alpha coefficient values between 0.54 and 0.75, and the test–retest fidelity value of 0.88 indicates the fidelity of the new 21-item form of the questionnaire (Cognitrom, 2024c).

**Table 1:** Attitude toward ChatGPT—16-item adapted scale.

V1 Item	V2 Item	Item	CC	AC	BC	M	SD	ITC
13	1	*ChatGPT is a tool that enhances my ability to develop academic projects and activities.* ChatGPT este un instrument care îmi îmbunătățește capacitatea de a dezvolta proiecte și activități academice.	0.71			4.38	1.27	0.28
14	2	*ChatGPT interface is user-friendly and easy to use.* Interfața ChatGPT este ușor de utilizat.	0.86			3.86	1.32	0.31
29	3	*Using ChatGPT in my academic activities allows me to explore different perspectives and approaches to address the contents of my subjects.* Folosirea ChatGPT în activitățile mele academice îmi permite să explorez diferite perspective și abordări.	0.77			3.21	1.09	0.37
30	4	*Frequent use of ChatGPT diminishes my abilities to think critically and solve problems independently*. Folosirea frecventă a ChatGPT îmi diminuează abilitățile de a gândi critic și de a rezolva probleme în mod independent.	0.71			2.45	1.12	0.43
31	5	*I am aware that not all answers provided by ChatGPT are correct.* Ştiu că nu toate răspunsurile oferite de ChatGPT sunt corecte.	0.86			2.77	1.44	0.35
33	6	*Irresponsible use of ChatGPT can diminish the development of my professional skills.* Utilizarea iresponsabilă a ChatGPT poate diminua dezvoltarea abilităților mele profesionale.	0.87			2.56	1.49	0.37
5	7	*I am attracted to the possibility of using ChatGPT to improve my academic productivity and efficiency.* Mă atrage posibilitatea de a folosi ChatGPT pentru a-mi îmbunătăți productivitatea și eficiența academică.		0.63		3.28	1.56	0.41
6	8	*I feel enthusiastic about using ChatGPT to seek solutions and answers to my academic concerns.* Simt entuziasm față de utilizarea ChatGPT pentru a căuta soluții și răspunsuri la preocupările mele academice.		0.72		3.57	1.22	0.48
9	9	*ChatGPT is a useful tool to understand and comprehend complex topics in my courses*. ChatGPT este un instrument util pentru a înțelege și înțelege subiecte complexe din cursurile mele.		0.81		3.49	1.34	0.31
21	10	*I dislike the idea that technology such as ChatGPT replaces certain human skills such as inferring, information seeking, analyzing, writing, etc.* Nu-mi place ideea că tehnologia precum ChatGPT înlocuiește anumite abilități umane, cum ar fi deducerea, căutarea de informații, analizarea, scrierea etc.		0.79		3.53	1.22	0.46
34	11	*I am concerned that frequent use of ChatGPT may limit my ability to think and solve problems independently.* Mă îngrijorează faptul că utilizarea frecventă a ChatGPT poate limita capacitatea mea de a gândi și de a rezolva probleme în mod independent.		0.74		3.13	2.04	0.48
35	12	*I am concerned that excessive use of ChatGPT will diminish my interest in researching and reading diverse sources of information.* Mă îngrijorează că utilizarea excesivă a ChatGPT îmi va diminua interesul pentru cercetarea și citirea diverselor surse de informații.		0.86		3.47	1.28	0.37
3	13	*I am open to use ChatGPT as part of my learning process at university.* Manifest deschidere față de posibilitatea de a folosi ChatGPT ca parte a procesului meu de învățare la universitate.			0.69	3.51	1.38	0.36
27	14	*It is not necessary to check the veracity of the information provided by ChatGPT because it always provides valid and reliable information.* Nu este necesar să verific veridicitatea informațiilor furnizate de ChatGPT deoarece oferă întotdeauna informații valide și de încredere.			0.83	2.88	1.22	0.43
37	15	*I use ChatGPT responsibly by not presenting technology-generated responses as if they were my own work product, without proper attribution.* Folosesc ChatGPT în mod responsabil, neprezentând răspunsuri generate de tehnologia AI ca și cum ar fi propriul meu produs.			0.74	2.44	1.19	0.47
40	16	*I strive to understand the limitations of ChatGPT and its potential to generate incorrect or biased responses, which motivates me to use it with caution and discernment.* Mă străduiesc să înțeleg limitările ChatGPT și potențialul său de a genera răspunsuri incorecte sau părtinitoare, ceea ce mă motivează să îl folosesc cu prudență și discernământ.			0.86	2.98	1.04	0.36

V1 item—original scale item (Acosta-Enriquez et al., 2024); V2 item—adapted scale item (translated into Romanian); CC—cognitive component; AC—affective component; BC—behavioral component; ITC—item total correlation.

## 3. Results

Starting from the general objective of the research (to analyze the attitude of students toward artificial intelligence—particularly toward ChatGPT—in relation to personality traits, coping strategies, and personal values), as there was no validated and standardized instrument on the Romanian population or Bucharest population to assess the attitude of students toward ChatGPT, the first specific objective of the research was to translate and adapt the scale designed by Acosta-Enriquez et al. (2024), which had statistically adequate psychometric characteristics, for students in Bucharest and also to evaluate the psychometric characteristics of the adapted scale.

The data analysis was performed using IBM SPSS Statistics 23 and AMOS 23.

The preliminary data analysis showed that there were no extreme values of the scale items, and, using a frequency analysis, the results of three subjects were excluded from the research, as they did not answer all the items of the scales. The normality of the distributions of the variables was investigated and validated by the skewness and kurtosis index values varying around 0, the result of the Shapiro–Wilk normality test (with values between 0.31 and 0.87 at a significance threshold of *p* < 0.001), and the Kolmogorov–Smirnov test (with values between 0.05 and 0.66 at a significance threshold of *p* > 0.05).

The residual values lay around the prediction line according to a random model, which meant that there was a linear relationship between the variable referring to students’ attitudes toward artificial intelligence (particularized to ChatGPT) and the investigated psychological variables (personality traits, coping strategies, and personal values).

The item analysis revealed that the difficulty index (between 0.2 and 0.5) and discrimination index (between 0.3 and 0.5) values indicated the scale items’ ability to correctly discriminate the subjects included in the research.

An item analysis also revealed that the item scores showed statistically significant (*p* < 0.05) positive intercorrelations (0.33 < r < 0.47). After investigating the internal consistency using the Cronbach’s Alpha coefficient of the 40 items of the original scale, the adapted scale was left with 16 items, as described in Table 1 (detailed results will be presented in the description of the scale’s fidelity).

The results were further processed in two steps:

- In the first stage, in order to check whether the scale keeps its fidelity (i.e., after translation and adaptation for students in Bucharest and after the first two items were excluded using the Delphi method, as they were very similar to the third one), its distribution of items (keeping only the items that had Cronbach’s alpha values greater than 0.7), and its three components/factors, an exploratory factor analysis (EFA) was applied with the principal component method (varimax rotation) (according to Costello & Osborne, 2005 and Velicer & Jackson, 1990 as cited in Popa, 2010).
- The second stage involved performing a confirmatory factor analysis to check the factor structure and whether the three factors hold.

After calculating the Cronbach’s Alpha coefficients, an exploratory analysis was performed, which showed an initial commonality of 1 for all items (and, after the rotation process, values between 0.63 and 0.86, indicating that the variables were well-represented by the respective factor model) and a saturation of at least 0.6 (this indicator being a criterion for item retention in accordance with the threshold accepted in the literature, according to Sava, 2004). The Kaiser–Meyer–Ohlin (KMO = 0.74) and Bartlett (χ^2^ = 572.29; *p* < 0.001) test values justified the approach of reducing the raw data.

Through performing an exploratory factor analysis (a principal components identification method, with varimax rotation and retention of factors with eigenvalues > 1.00), three factors were identified as similar to the initial scale. The adapted scale items loaded (with an eigenvalue of 1.05) on three factors that explained 53% of the variance, the remaining up to 100% being unexplained by this model. The variance explained by each factor was distributed as follows: factor 1—20.25%; factor 2—12.74%; and factor 3—20.01%. After the rotation, an eigenvalue of 1.28 was obtained, and the variance explained by each factor was redistributed as follows: factor 1—21.34%; factor 2—14.79%; and factor 3—16.87% (this factor lost saturation in favor of the others). However, the total variance explained still represented 53% (data are presented in Table 2).

The names of the three factors identified by the factorial model of the first form of the scale of evaluation of students’ attitudes toward ChatGPT (designed by Acosta-Enriquez et al., 2024) remained the same in the adapted scale (first, the cognitive component; second, the affective component; third, the behavioral component), but their definition and conceptualization were extended, taking into account the content of the items.

Thus, the adapted scale that evaluates the students’ attitude toward ChatGPT contains the following conceptualizations of the factors/components:

1. The cognitive component describes students’ beliefs about ChatGPT both from a favorable perspective (in the case of high scores, they considered it a tool that improves their ability to develop projects and academic activities, allows them to explore different perspectives and approaches, and is easy to use) and from the perspective of concerns and mistrust (in the case of low scores, they considered that the frequent use of ChatGPT diminishes their ability to think critically and solve problems independently, and they are aware that not all answers provided by ChatGPT are correct and its irresponsible use may diminish the development of their professional skills);
2. The affective component describes students’ preferences both from a favorable perspective (in the case of high scores, regarding the possibility of using ChatGPT in order to improve their academic productivity and efficiency, to seek solutions and answers to their academic concerns, and to understand complex topics in their courses), as well as from the perspective of worries and mistrust (in the case of low scores, regarding the idea that technologies like ChatGPT replace certain human skills, such as deduction, searching for information, analyzing, and writing, they could limit the ability to think and solve problems independently, and their excessive use would decrease their interest in researching and reading various sources of information);
3. The behavioral component describes some behaviors both from the favorable perspective (in the case of high scores, using ChatGPT as part of the university learning process, the students showing a high level of trust and considering that it is not necessary to check the accuracy of the information provided by ChatGPT because it always provides valid information), as well as from the perspective of concerns and mistrust (in the case of low scores, specifying that they use ChatGPT responsibly, not presenting AI-generated answers as their own, understanding ChatGPT’s limitations and its potential to generate incorrect or biased responses, which motivates them to use it with caution and discernment).

A confirmatory factor analysis (CFA) was performed to check the validity of the three-factor structure identified in the exploratory analysis.

**Figure 1:**
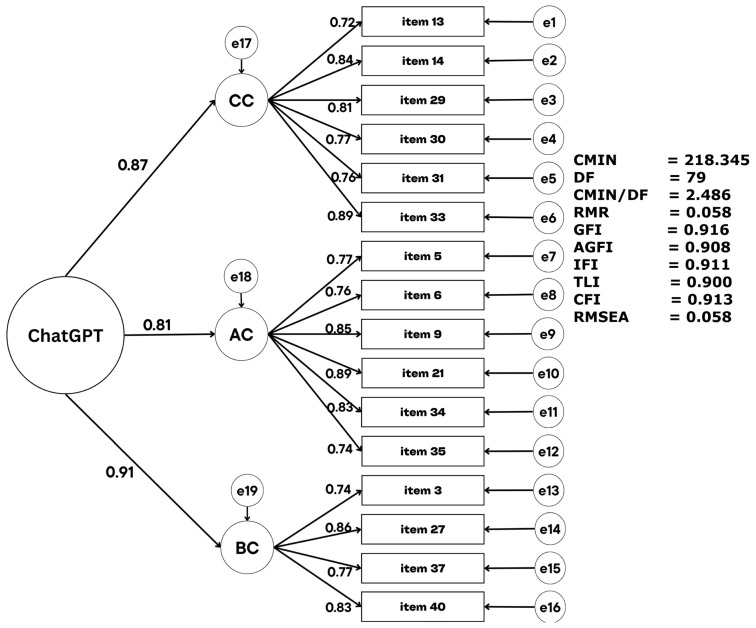
Standardized CFA for the three-factor 16-item structure model.

The obtained results showed that the proposed model contains the three factors (cognitive, affective, and behavioral components) as latent variables, with their items acting as observed variables. A second-order factor was also included to represent the general use of the adapted ChatGPT scale structural model (Figure 1).

The CFA results indicated that the proposed model was appropriate: χ^2^(79) = 218.345, *p* < 0.001; CMIN/DF = 2.486; CFI = 0.911; TLI = 0.900; RMSEA = 0.058 (90% CI: 0.50–0.065). All standardized factor loadings were statistically significant (*p* < 0.001) and ranged from 0.434 to 0.728, exceeding the minimum threshold of 0.4 (Hair et al., 2014).

The loadings of the second-order factor were also strong, with their standardized coefficients being 0.87 for CC, 0.81 for AC, and 0.91 for BCT, but the first-order factors were better represented by the adapted ChatGPT scale.

The scaling of the items was maintained: students self-assessed how they characterized the behaviors described by the items using five steps (1—never, 5—always).

The adapted scale allowed the calculation of an overall score but also a subscale/component. Since the items were formulated as affirmative statements, in order not to affect the accuracy of the results and to relate the above-average score to a favorable, open attitude toward ChatGPT (referring to all of the three components: cognitive, affective, and behavioral) and the low scores to an attitude of distrust and concern, certain items, marked in the table with „–”, were scored inversely (for example, for item „10”, a „never” response will be scored with 5 instead of 1 and an „always” with „1” instead of „5”; see Table 3).

The results were analyzed at a general level, and calibration on the subjects of this research was carried out (taking into account the fact that the distribution of the raw results was very close to the normal distribution) on normalized classes and involved three of these classes/levels corresponding to the three attitudes toward ChatGPT (distrustful/fearful, medium/cautious, and positive/open), thus obtaining the global standard. At the same time, the existence of three factors/components with relative autonomy in terms of investigating resilience allowed for the use of a specific/independent benchmark for the strict assessment of the construct associated with the respective dimension (Table 4).

- The psychometric characteristics of the adapted scale—fidelity and validity

The analysis of the Cronbach’s Alpha coefficient of the adapted scale (after it was observed that by eliminating any item, there was no rise in the values of any coefficient for each subscale, and 16 items remained from the original 40) showed a value of 0.86, and the values between 0.81 and 0.91 of the Cronbach’s Alpha coefficient for the three components revealed that both the scale and the components had a statistically adequate internal consistency of the items (Popa, 2010) and denoted the fidelity of the scale. The results shown in Table 5 (0.73 > r > 0.86) prove the test–retest reliability of the adapted scale of students’ evaluated attitudes toward ChatGPT (being reapplied after three weeks on 50 students).

Content validation was ensured through translation of the scale by authorized expert translators for the field of psychology, as well as use of the Delphi method (five experts evaluated whether each item of the scale was translated correctly, maintaining the meaning to be correctly understood by the research subjects).

The second objective involved investigating the relationship between students’ attitudes toward ChatGPT and certain psychosocial variables (e.g., personality traits, coping strategies, and students’ personal values) to identify psychosocial approaches for students’ effective reporting toward this AI product in the university environment.

The primary data analysis showed that 85.53% of the research subjects used ChatGPT at least once, of which, and taking into account the benchmark obtained from the adaptation of the scale (Acosta-Enriquez et al., 2024) on students from Bucharest, Romania, 24.11% (representing 27.86% of the total research subjects) have a positive/open attitude toward ChatGPT, 36.74% have a cautious attitude (representing 42.45% of the total research subjects), and 25.68% (representing 29.67% of the total research subjects) have a distrustful/fearful attitude.

The results indicated statistically significant positive correlations (*p* < 0.001) between the students’ open attitude toward ChatGPT and certain personality traits: extraversion (r = 0.56) and autonomy (r = 0.64). The analysis of the results revealed statistically significant positive correlations (*p* < 0.001) between the students’ favorable attitudes toward ChatGPT and the following frequently used effective coping strategies/styles: focus on planning (r = 0.61), positive focus (r = 0.58), putting into perspective (r = 0.54), and positive reappraisal (r = 0.48).

The results showed that there are also statistically significant negative correlations (*p* < 0.01) between the open attitude toward ChatGPT and a series of coping styles, such as rumination (r = −0.56), catastrophizing (r = −0.62), and blaming others (r = −0.57).

Additionally, the open attitude of students toward ChatGPT correlated positively and in a statistically significant (*p* < 0.001) manner with the following personal values: professional recognition (r = 0.63), autonomy (r = 0.67), and challenge (r = 0.56). They also correlated negatively with the value of safety (r = −0.69).

The analysis of the results obtained, taking into account the three components of students’ attitude toward ChatGPT (cognitive, affective, and behavioral), shows that each of them correlates positively or negatively, statistically significantly, with a series of research variables. Thus, we conclude the following:

The cognitive component correlates statistically significantly (*p* < 0.01) with a series of personal values: professional recognition (r = 0.69), autonomy (r = 0.63), challenge (r = 0.55), and value of safety (r = −0.64), with coping strategies centered on rumination (r = −0.54), catastrophizing (r = −0.66), and blaming others (r = −0.53).

The affective component correlates statistically significantly (*p* < 0.01) with a series of coping strategies: focus on planning (r = 0.61), positive focus (r = 0.58), putting into perspective (r = 0.52), positive reappraisal (r = 0.49), rumination (r = −0.51), catastrophizing (r = −0.62), and blaming others (r = −0.58).

The behavioral component correlates statistically significantly (*p* < 0.01) with a series of personality traits: extraversion (r = 0.71) and autonomy (r = 0.65); with the personal value autonomy (r = 0.62); and with coping strategies centered on focus on planning (r = 0.66), putting into perspective (r = 0.52), and positive reappraisal (r = 0.49).

The size of the effect of each statistically significant positive or negative correlation between the different variables was calculated using the r^2^ coefficient. The values of this coefficient were between 0.23 and 0.50, above the limit of 0.13, thus indicating a medium-to-high level of association between the variables (Cohen, 2004 as cited in Hair et al., 2014), which, beyond the statistical level, adequately reflected reality.

## 4. Discussion

The present research was carried out on the basis of a number of national government initiatives in the field of AI, as well as of international studies on the relationship between the attitude toward AI (and, in particular, toward ChatGPT) and various psychosocial variables associated with performance.

A starting point for this research was the issuance of the „National Strategy in the Field of Artificial Intelligence 2024–2027” by the Ministry of Research, Innovation, and Digitization; this document, under General Objective 3 (OG3), „Development of the National System of Research–Development–Innovation in the Field of AI”, contains the Specific Objective 3.1 (OS3.1), „Development of Fundamental and Applied Scientific Research Specific to the AI Domain as well as at an Interdisciplinary Level” Government of Romania (2024).

Another aspect that determined the elaboration of this study was the organizing (on 30 September 2024, Parlamentul României SENAT, 2024) by two commissions of the Romanian Senate of the international conference entitled „Information Technology and the Health of Young People. One Click away from the Digital Pandemic”. In that context, specialists from various fields highlighted the challenges that they, the students, the students’ families, and teachers face in terms of the influence of AI on the development of young people.

Furthermore, this research was supported by the analysis of some concepts from the specialized literature that associated the use of AI (and, in particular, ChatGPT) by students with positive effects on university activities (Nemt-allah et al., 2024; Hartley et al., 2024), as well as from some studies that showed both the positive effects and the risks associated with the acceptance of AI in an educational context (Abdaljaleel et al., 2024; Choi et al., 2024; Sallam et al., 2024; Hutson et al., 2022; Y. M. Wang et al., 2022).

In accordance with the national and international premises, the present research had as its general objective the investigation of Bucharest students’ attitudes toward ChatGPT in relation to their personality traits, coping strategies, and personal values to identify psychosocial approaches for students’ effective reporting toward this AI product, so it would be a factor of progress in university activities.

As there was no instrument validated and calibrated on Romanian students, the scale built by Acosta-Enriquez et al., which included the concepts of Mitcham (1994) as cited in Acosta-Enriquez et al. (2024) regarding the three components of the attitude toward technology (cognitive, affective, and behavioral) and the deterministic integrated model of Svenningsson et al. (2022) as cited in Acosta-Enriquez et al. (2024), was adapted using 508 students from eleven faculties from Bucharest, Romania.

Following the item analysis, the adapted scale was reduced to 16 items (from 40 in the original form), and following the factor analysis, the three-factor structure was maintained, given the results of the confirmatory factor analysis.

The names of the three factors identified by the factorial model of the original form of the scale remained the same in the case of the adapted scale, but the definition and conceptualization were expanded, considering the content of the items. Additionally, the standard of the adapted scale was modified to reflect the results of the students in Bucharest.

The appropriate values of the Cronbach’s Alpha coefficient for the subscales and the total scale indicate the fidelity of the adapted scale, and the content validity was evaluated using the Delphi method, which proves that the adapted scale has adequate psychometric characteristics from a statistical point of view and can be used with confidence to evaluate the attitude of Bucharest students toward ChatGPT.

The present research also brings, as a novelty, the investigation of students’ attitude toward ChatGPT expressed also in percentage; thus, it is shown that 85.53% of the 508 students of the faculties of the universities in Bucharest, Romania, who constituted the research subjects have used ChatGPT at least once; of these, 24.11% have a positive/open attitude toward ChatGPT, while 36.74% have a cautious attitude and 25.68% have a distrustful/fearful attitude.

International studies on the analysis of attitude toward ChatGPT show that over 50% of higher education students from several European countries (France, Germany, Italy, Poland, Spain, Sweden, The Netherlands, Austria, Germany, France, Spain, Italy, and the United Kingdom) have a positive attitude toward AI chatbots, such as ChatGPT (Scantamburlo et al., 2024). Many students perceive ChatGPT as a useful tool for brainstorming, summarizing texts, explaining complex concepts, and supporting academic writing (Sublime & Renna, 2024).

At the international level, a study conducted between 2023 and 2024 on 23,218 students from 109 countries showed that most students have a positive perception of ChatGPT, and 70% of students stated that they find ChatGPT interesting to use in academic activities, using it for brainstorming, summarizing, writing, and clarifying complex information (Ravšelj et al., 2025).

While students from various countries show a positive/open attitude toward ChatGPT in a significantly higher percentage, Romanian students are in a phase of exploration and accommodation, and the data provided by this research complete the international picture regarding the percentage of Romanian students regarding their attitude toward ChatGPT as an AI chatbot.

The relationship between the attitude of Bucharest students toward ChatGPT and certain psychosocial variables (personality traits, coping strategies, and personal values) was investigated within the second objective of the research.

The results showed that students characterized by extraversion and those who have a high level of autonomy express their own opinions and act on them tend to have an open attitude toward ChatGPT. Along the same direction of analysis, some studies have identified a positive association between extraversion as a personality trait of students and acceptance of technology (Kaya et al., 2022), while others have shown a negative association between the two, indicating that the more introverted students are, the more inclined they are to use AI chatbots (Schepman & Rodway, 2022).

At the same time, students who have personal values such as autonomy (as a resource of freedom in action), professional recognition (manifested as the need for recognition and respect of competence in their own field of activity), and challenge (understood as a willingness to be involved in risky and complex activities but with a potential positive finality on their own development) tend to have an open attitude toward ChatGPT.

Regarding the coping strategies used in managing difficult situations, the results of this research revealed that students with a positive/open attitude toward ChatGPT frequently use coping strategies such as focusing on planning, positive focus, putting into perspective, and positive re-evaluation—aspects associated with the success of carried-out activities. In a similar direction, there are studies in the specialized literature that associate coping strategies with students’ psychological well-being (Moreno-Montero et al., 2024; Sumin et al., 2024), as well as studies that relate attitudes toward chatbots with employees’ coping strategies but also associate them with the fear of losing their jobs and being replaced by artificial intelligence chatbots (Y. H. Chen et al., 2025).

The results of the research also indicated the fact that students characterized by introversion (manifested by closing in on themselves, not expressing opinions in various contexts, etc.) tend to show a distrustful/fearful attitude, including toward ChatGPT.

Also, students who more frequently use ineffective coping styles (such as rumination, catastrophizing, and blaming others), who have a low level of autonomy, and who have safety as their predominant value (expressed by orientation toward activities with predictable development and outcome) tend to show a distrustful/fearful attitude, including toward ChatGPT.

The relationship between ChatGPT use and students’ values has been studied before, but not by analyzing the types of values (Cognitrom, 2024c) described in this research, but by analyzing either morality as a moderating variable in students’ compliance with academic ethics, showing that students’ personal morality leads them to experience regret when they violate academic integrity through unethical use of ChatGPT (Elbaz et al., 2024), or by analyzing anxiety about using AI in order not to violate ethical standards (Zhu et al., 2024).

The present study identified relationships between the students’ attitudes toward ChatGPT and their personality traits, coping strategies, and personal values in a correlational manner, thus indicating an association between the variables. More detailed statistical analyses showed that the psychosocial variables that were associated with students’ attitudes toward ChatGPT at the general level also correlated with its three components (cognitive, affective, and behavioral).

## 5. Conclusions

The general objectives of this research were to investigate the attitude of Bucharest students toward ChatGPT and the correlated psychosocial variables and to identify psychosocial approaches for students’ effective reporting toward this AI product; the two specific objectives were achieved.

The way students relate to technology, AI, or, in particular, ChatGPT, is associated with their certain personality traits, personal values, and coping strategies (as the results of this research also showed) but can also be associated with other psychosocial characteristics that will be investigated in future studies, such as formal and informal learning of the ethical use of AI.

Based on this conception, as well as theories from the specialized literature such as the cognitive load theory, which provides explanations regarding the analysis of the benefits and cognitive challenges associated with technological assistance in academic tasks (O. Chen, 2015; Lo, 2023), the authors of this research propose a series of means that have been developed to stimulate the open but ethical reporting by students on ChatGPT.

Among the methods/approaches for managing the relationships with ChatGPT in a university environment, the following can be listed:

- Information on the benefits of appropriate use (e.g., ChatGPT provides general information in the form of quick summaries, supports overcoming writer’s block when running out of ideas, etc.) and information on the involved risks (exercises to check some information provided by ChatGPT and concrete examples of errors in the information transmitted by ChatGPT).
- Recalling ethical principles in university activities and providing information about the emergence of programs for detecting materials that are not designed by humans but are designed based on AI and presenting examples of such programs: ZeroGPT (2025), Zero GPT Detector (2025), and so on.
- Emphasizing the importance of developing critical thinking, innovation, and personal creativity in writing papers.
- Mentioning effective time or stress management strategies (in pressure situations determined, for example, by a deadline, there may be a tendency to unethically use ChatGPT).

Therefore, one of the directions of action in the field of psychology could be the elaboration of effective programs for time and coping management strategies, such that students relate to ChatGPT cautiously without developing addiction or fear and recognize the benefits brought to the evolution of education.

This conception was based on studies by many authors who showed that beyond ethical challenges or those related to the clarity of the information presented, ChatGPT has the potential to improve teaching and learning and can be a valuable tool for both students and instructors, providing a starting point for creating course programs, teaching materials (providing students with an interactive and personalized learning environment), and assessment tasks (Lo, 2023; Nemt-allah et al., 2024; Hartley et al., 2024).

### Limitations and Future Study

Although the general objective was achieved within the research, and the adapted scale has statistically adequate psychometric qualities and is based on a significant number of subjects (N = 508) coming from eleven faculties from Bucharest, the research also has a series of limitations; however, it can serve as a starting point for other, more extensive research, both in terms of the number of subjects and the research design. The main limitations of the research are that only self-reported data were used and that the general attitudes toward technology were not considered, which are variables that will be analyzed in future studies.

The future directions of action will involve expanding the number of subjects, selecting them from universities from other areas of the country (for validating the scale on the Romanian population), and applying the adapted scale to them in order to investigate their psychometric qualities. Also, studies will continue through the analysis of convergent and divergent validity, and concurrent validity compares the results with those of other similar or distinct scales that would also contain other subscales that add to the approach to and evaluation of the attitude toward ChatGPT.

These directions are in agreement with a number of conceptions in the specialized literature that have associated the use of AI (and, in particular, of ChatGPT) by students with certain positive effects in learning and reporting to university activities, including help with academic writing, support for various academic tasks (Nemt-allah et al., 2024), and writing support through idea generation, paraphrasing, and the development of counterarguments (Hartley et al., 2024). A new direction for future research could be the correlated analysis but also the comparison between students using ChatGPT with intrinsic motivation (assessed through the cognitive and affective components of the adapted Acosta-Enriquez et al., 2024 scale) and those who also have extrinsic motivation, assessed through investigating the attitude of the social group they belong to, taking into account the self-determination theory (Ng et al., 2012; Annamalai et al., 2025).

Another direction that can be addressed in future studies could target the association between students’ attitude toward ChatGPT and attitude toward technology acceptance, taking into account the principles of the TAM model (Davis, 1986; Yilmaz et al., 2023; Zhai et al., 2024) and a series of studies that have measured the level of acceptance of AI technology in educational contexts, taking into account both the positive effects and risks (Sallam et al., 2023; Abdaljaleel et al., 2024).

In conclusion, the present research brings the following as novelties:

(a) A translated, validated, and calibrated scale (The Attitude toward ChatGPT Identification Scale—Acosta-Enriquez et al., 2024) on students from Bucharest.
(b) It offers concrete percentage data on the attitudes of students from Bucharest toward ChatGPT.
(c) The fact that it analyzes types of personal values of the students that have not been investigated before in relation to the attitude toward an AI product.
(d) The fact that it proposes several concrete steps for the efficient management of students’ relationships with ChatGPT.

The final objective of this research was to draw a series of directions, both for the extensive research of students’ attitudes toward ChatGPT and the associated variables, as well as for studies carried out together with other institutions interested in the subject, with the aim of identifying approaches for the effective management of reporting on ChatGPT through an open attitude, in which, beyond the risks, the benefits brought to the evolution of education are recognized.

## Figures and Tables

**Table 2:** The Attitude Toward ChatGPT—16 Items Adapted Scale factor loading to the factorial model (after the exploratory factorial rotation with the method of main components—varimax rotation).

Items	Factor Loading			Communality
	F1	F2	F3	
Item 1	0.71			0.78
Item 2	0.86			0.83
Item 3	0.77			0.63
Item 4	0.71			0.71
Item 5	0.86			0.75
Item 6	0.87			0.77
Item 7		0.63		0.65
Item 8		0.72		0.81
Item 9		0.81		0.74
Item 10		0.79		0.78
Item 11		0.74		0.73
Item 12		0.86		0.86
Item 13			0.69	0.75
Item 14			0.83	0.81
Item 15			0.74	0.77
Item 16			0.86	0.82
Variance	21.34%	14.79%	16.87%	53%

**Table 3:** Internal consistency of the adapted scale’s subscales and the distribution of items on subscales.

Subscales	Number of Items	Cronbach’s α Coefficients	Items
Cognitive component	6	0.87	1, 2, 3, −4, −5, −6
Affective component	6	0.81	7, 8, 9, −10, −11, −12
Behavioral component	4	0.91	13, 14, 15, −16

**Table 4:** Adapted scale’s benchmark and its subscales.

Attitude Toward ChatGPT and Its Subscales	Level Distrustful/Fearful	Medium/Cautious	Positive/Open
Attitude toward ChatGPT—global score	Under 37	38–54	Over 55
Cognitive component	Under 10	11–20	Over 21
Affective component	Under 9	10–21	Over 22
Behavioral component	Under 7	8–15	Over 16

**Table 5:** Test–retest reliability of the adapted scale.

Pearson Correlation Coefficient (r)	Adapted Scale	Cognitive Component	Affective Component	Behavioral Component
Adapted scale	0.81 **			
Cognitive component		0.83 **		
Affective component			0.86 **	
Behavioral component				0.73 **

** Significant correlations at level 0.01 bilateral.

## Data Availability

The original contributions presented in this study are included in the article. Further inquiries can be directed at the corresponding author.
